# Differential expression of angiotensin-converting enzyme-2 in human paranasal sinus mucosa in patients with chronic rhinosinusitis

**DOI:** 10.1017/S0022215121001225

**Published:** 2021-04-30

**Authors:** T Kawasumi, S Takeno, M Nishimura, T Ishino, T Ueda, T Hamamoto, K Takemoto, Y Horibe

**Affiliations:** Department of Otorhinolaryngology, Head and Neck Surgery, Graduate School of Biomedical Sciences, Hiroshima University, Hiroshima, Japan

**Keywords:** Angiotensin-Converting Enzyme 2, Coronavirus, Eosinophils, Respiratory Epithelium, SARS-CoV-2, Sinusitis, Tumor Necrosis Factor-Alpha

## Abstract

**Objective:**

Severe acute respiratory syndrome coronavirus-2 uses angiotensin-converting enzyme-2 as a primary receptor for invasion. This study investigated angiotensin-converting enzyme-2 expression in the sinonasal mucosa of patients with chronic rhinosinusitis, as this could be linked to a susceptibility to severe acute respiratory syndrome coronavirus-2 infection.

**Methods:**

Ethmoid sinus specimens were obtained from 27 patients with eosinophilic chronic rhinosinusitis, 18 with non-eosinophilic chronic rhinosinusitis and 18 controls. The angiotensin-converting enzyme-2 and other inflammatory cytokine and chemokine messenger RNA levels were assessed by quantitative reverse transcription polymerase chain reaction. Angiotensin-converting enzyme-2 positive cells were examined immunohistologically.

**Results:**

The eosinophilic chronic rhinosinusitis patients showed a significant decrease in angiotensin-converting enzyme-2 messenger RNA expression. In the chronic rhinosinusitis patients, angiotensin-converting enzyme-2 messenger RNA levels were positively correlated with tumour necrosis factor-α and interleukin-1β (r = 0.4971 and r = 0.3082, respectively), and negatively correlated with eotaxin-3 (r = −0.2938). Angiotensin-converting enzyme-2 immunoreactivity was mainly localised in the ciliated epithelial cells.

**Conclusion:**

Eosinophilic chronic rhinosinusitis patients with type 2 inflammation showed decreased angiotensin-converting enzyme-2 expression in their sinus mucosa. Angiotensin-converting enzyme-2 regulation was positively related to pro-inflammatory cytokines, especially tumour necrosis factor-α production, in chronic rhinosinusitis patients.

## Introduction

Severe acute respiratory syndrome coronavirus-2 (SARS-CoV-2) emerged in December 2019 in China, and has caused a global health emergency.^1^ This new infection, named coronavirus disease 2019 (Covid-19), presented a huge burden to healthcare facilities, especially when it occurred alongside acute respiratory distress syndrome.^2,3^

Accumulating epidemiological data indicated that the major transmission route of SARS-CoV-2 is airborne droplets in the respiratory tract.^4^ During the early phase of the SARS-CoV-2 outbreak, higher rates of nosocomial spread were observed, possibly due to a high viral load in upper respiratory tract mucosa.^3,5^ A recent report revealed that the nasal respiratory epithelium had a higher expression of SARS-CoV-2 entry genes than that of the trachea or lungs.^6^ It was speculated that the nose and paranasal sinuses were important portals for the initial SARS-CoV-2 infection, and served as a key reservoir for viral spread throughout the respiratory tract.^7^

The pandemic has highlighted the importance of angiotensin-converting enzyme-2 (ACE2), which is the binding site for the transmembrane S1 spike glycoprotein of SARS-CoV-2, and of transmembrane serine protease 2 (TMPRSS2), which primes the S2 subunit prior to cell membrane fusion. Angiotensin-converting enzyme-2 is a homolog of angiotensin-converting enzyme, which is a crucial component of the renin–angiotensin system.^8^

In human homeostasis, ACE2 plays important roles in several cardiovascular and immune pathways, with protective effects against organ damage. Angiotensin-converting enzyme-2 is expressed in the vascular, digestive, renal and respiratory systems.^9,10^ However, the precise physiological role of ACE2 in the human respiratory tract has not been identified.

We hypothesise that ACE2 distribution in sinonasal mucosa is affected by inflammation. As this may influence susceptibility to SARS-CoV-2, we investigated the gene expression and protein distribution in the sinus mucosa of patients with chronic rhinosinusitis and of controls. In Japan, chronic rhinosinusitis is classified as eosinophilic chronic rhinosinusitis or non-eosinophilic chronic rhinosinusitis. Notably, eosinophilic chronic rhinosinusitis with nasal polyps is characterised by eosinophilic inflammation associated with elevated levels of Th2 cytokines.^11,12^

## Materials and methods

### Study design

The study was cross-sectional. Forty-five chronic rhinosinusitis patients who underwent endoscopic sinus surgery were included. The diagnosis of chronic rhinosinusitis was based on: the patient's history, clinical symptoms, endoscopic findings and computed tomography (CT) findings. None of the study patients had received topical or systemic steroids during the four weeks prior to the surgery. The CT images were graded using the Lund–Mackay system (as cited in Lund and Kennedy^13^).

We divided the chronic rhinosinusitis patients into an eosinophilic chronic rhinosinusitis group (*n* = 27) and a non-eosinophilic chronic rhinosinusitis group (*n* = 18), based on the Japanese Epidemiological Survey of Refractory Eosinophilic Chronic Rhinosinusitis criteria.^14^ All of the eosinophilic chronic rhinosinusitis patients had nasal polyps with intense tissue eosinophilia (70 or more cells per high power field) in the submucosa. The control group were 18 age-matched individuals with no clinical or radiological evidence of rhinosinusitis.

### Reverse transcription polymerase chain reaction analysis

Tissue samples were taken from the ethmoid sinus and nasal polyps (if present). The specimens were either immersed in RNAlater solution (Ambion, Austin, Texas, USA) for reverse transcription polymerase chain reaction analysis or fixed in 4 per cent paraformaldehyde for immunohistochemistry. A quantitative polymerase chain reaction analysis was performed on an ABI Prisms 7300 system (Applied Biosystems, Foster City, California, USA). Cellular RNA was isolated using RNeasy Mini Kits (Qiagen, Valencia, California, USA). Total RNA was then reverse-transcribed to complementary DNA using a High Capacity RNA-to-cDNA Kit (Applied Biosystems). Gene expression was measured on a real-time polymerase chain reaction system using TaqMan Gene Expression Assays (Life Technologies, Carlsbad, California, USA).

The polymerase chain reaction primers used were specific for: ACE2 (Hs01085333_m1), tumour necrosis factor-α (Hs99999043_m1), interleukin-1β (Hs01555410_m1), eotaxin (Hs00237013_m1), eotaxin-3 (Hs00171146_m1) and granulocyte-macrophage colony-stimulating factor (‘GM-CSF’) (Hs00929873_m1). Primers for glyceraldehyde 3-phosphate dehydrogenase (GAPDH) (Hs03929097_g1) served as a reference.

The polymerase chain reaction cycles were run in triplicate for each sample. Amplifications of the polymerase chain reaction products were quantified by the number of cycles and analysed using the comparative cycle threshold methods (2^-ΔΔ^Ct). Target gene expression was presented as relative rate compared to the expression of the reference gene (ratio: target gene/GAPDH expression).

### Immunohistochemistry

Angiotensin-converting enzyme-2 protein distribution was assessed using anti-human monoclonal antibody (number 66699-1-Ig; Proteintech, Rosemont, Illinois, USA). Approximately 5 μm thick cryostat sections were immersed in HistoVT One (Nacalai Tesque, Kyoto, Japan) at 70°C for 40 minutes. The slides were incubated overnight at 4°C with the primary antibodies. Colour development used the streptavidin-biotin amplification technique (ChemMate EnVision Kit; Dako, Glostrup, Denmark). Peroxidase activity was visualised using diaminobenzidine solution. Sections were counterstained with haematoxylin. Control specimens without the primary antibody were used to verify that non-specific binding was not detectable. Consecutive sections were stained with haematoxylin and eosin to assess mucosal pathology and the degree of eosinophil infiltration.

### Ethical standards

All procedures contributing to this work complied with the ethical standards of the Helsinki Declaration. The study protocol was approved by the Institutional Review Board at the Hiroshima University School of Medicine (approval number: Hi-136-2). Written informed consent was obtained from all patients and control subjects prior to their participation.

### Data analysis

Statistical methods included the Kruskal–Wallis and Mann–Whitney U tests for between-group analysis. The chi-square test was used to compare qualitative data. Correlation co-efficients were calculated by the Spearman method. *P*-values of less than 0.05 were considered statistically significant.

## Results

We divided the 45 chronic rhinosinusitis patients into eosinophilic chronic rhinosinusitis (*n* = 27) and non-eosinophilic chronic rhinosinusitis (*n* = 18) groups based on the Japanese Epidemiological Survey of Refractory Eosinophilic Chronic Rhinosinusitis criteria (Table 1). There were no significant differences in age, gender, body mass index or smoking status between the groups or controls (*n* = 18). The eosinophilic chronic rhinosinusitis group had a higher incidence of asthma, and blood and tissue eosinophils (*p* < 0.01, *p* < 0.001 and *p* < 0.001, respectively). The group CT scores were 17.1 ± 6.3 for the eosinophilic chronic rhinosinusitis group and 7.29 ± 5.3 for the non-eosinophilic chronic rhinosinusitis group (*p* < 0.001).

**Table 1.** Demographics and clinical background of study population

Parameter	Control group	Chronic rhinosinusitis groups	
		Non-eosinophilic group	Eosinophilic group
Number of participants	18	18	27
Males/females (*n*)	10/8	7/11	14/13
Age (mean ± SD; years)	43.5 ± 20.2	53.6 ± 15.8	52.3 ± 12.7
Allergic rhinitis (*n* (%))	12 (66.6)	11 (61.1)	17 (62.9)
BMI (mean ± SD; kg/mm^2^)	22 ± 2.4	21.7 ± 3.0	22.3 ± 3.4
Bronchial asthma (*n* (%))	1 (5.5)	3 (16.6)	14 (51.8)*
Smoking status (never smoked / current or former smoker; *n*)	16/2	14/4	18/9
Blood eosinophils (mean ± SD; % of blood)	3.0 ± 2.4	2.8 ± 2.5	7.5 ± 3.5^†^
CT score (mean ± SD)	–	7.29 ± 5.3	17.1 ± 6.3^‡^
Tissue eosinophils (mean ± SD; cells per HPF)	6.7 ± 7.4	14.8 ± 18.5	154.6 ± 103.2^†^

*p < 0.01; ^†^*p* < 0.001 versus the other groups; ^‡^*p* < 0.001 versus the non-eosinophilic chronic rhinosinusitis group. SD = standard deviation; BMI = body mass index; CT = computed tomography; HPF = high power field (×400)

### Target gene expression in sinus mucosa and nasal polyps

**Fig. 1.**
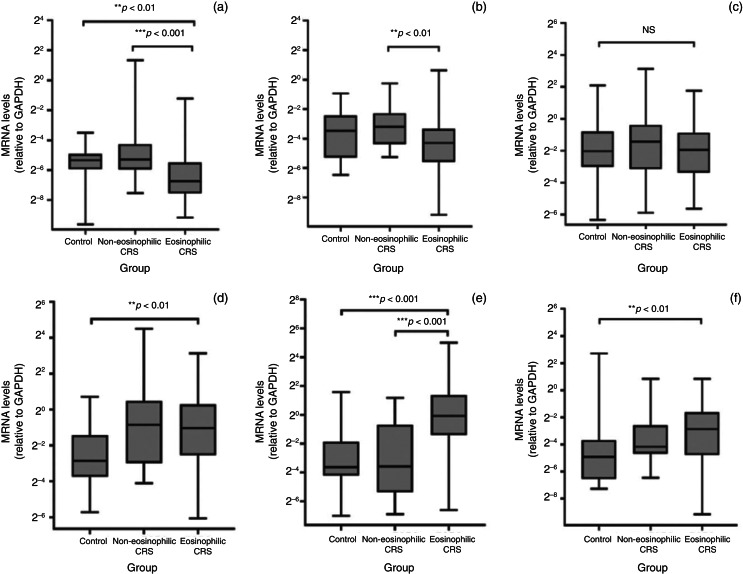
Comparison of messenger RNA (mRNA) expression in paranasal sinus mucosa from the controls, and the non-eosinophilic and eosinophilic chronic rhinosinusitis (CRS) patients, as detected by reverse transcription polymerase chain reaction. (a) Angiotensin-converting enzyme-2, (b) tumour necrosis factor-α, (c) interleukin-1β, (d) eotaxin, (e) eotaxin-3 and (f) granulocyte-macrophage colony-stimulating factor messenger RNA levels were quantitatively normalised to the glyceraldehyde 3-phosphate dehydrogenase (GAPDH) messenger RNA levels. Centre lines indicate median values, boxes represent interquartile ranges and error bars show overall ranges. **p < 0.01; ***p < 0.001; NS = not significant

Angiotensin-converting enzyme-2 messenger RNA was detected in all of the sinus specimens from the three groups. We detected a significant difference in ACE2 messenger RNA levels between the groups (Figure 1). The eosinophilic chronic rhinosinusitis patients had a significant down regulation of ACE2 messenger RNA expression compared to the non-eosinophilic chronic rhinosinusitis and controls. The non-eosinophilic chronic rhinosinusitis group had a significant increase in tumour necrosis factor-α (TNF-α) messenger RNA expression compared to the eosinophilic chronic rhinosinusitis group. The eosinophilic chronic rhinosinusitis patients had upregulations of eotaxin, eotaxin-3 and granulocyte-macrophage colony-stimulating factor messenger RNA expression compared to the control subjects. In contrast, the non-eosinophilic chronic rhinosinusitis patients tended to show higher ACE2, TNF-α and interleukin (IL)-1β messenger RNA levels than controls, but the difference was not significant.

**Fig. 2.**
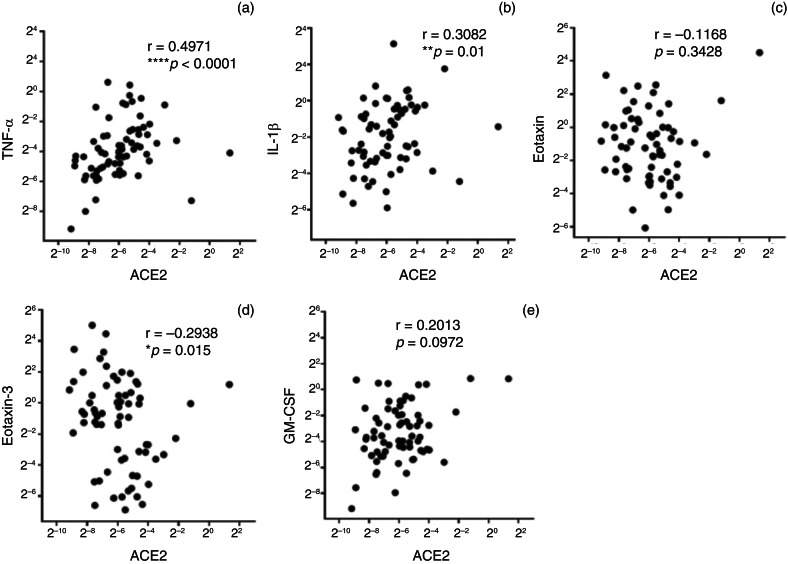
Correlation of messenger RNA expression levels between angiotensin-converting enzyme-2 (ACE2) and a panel of inflammatory cytokines and chemokines in sinus mucosa from chronic rhinosinusitis patients. *p < 0.05; **p < 0.01; ****p < 0.0001. TNF-α = tumour necrosis factor-α; IL-1β = interleukin-1β; GM-CSF = granulocyte-macrophage colony-stimulating factor

In chronic rhinosinusitis patients (Figure 2), TNF-α and IL-1β messenger RNA levels were positively correlated with ACE2 (r = 0.4971 for TNF-α and r = 0.3082 for IL-1β). In contrast, there was a negative correlation between ACE2 and eotaxin-3 (r = −0.2938).

### Angiotensin-converting enzyme-2 protein distribution

**Fig. 3.**
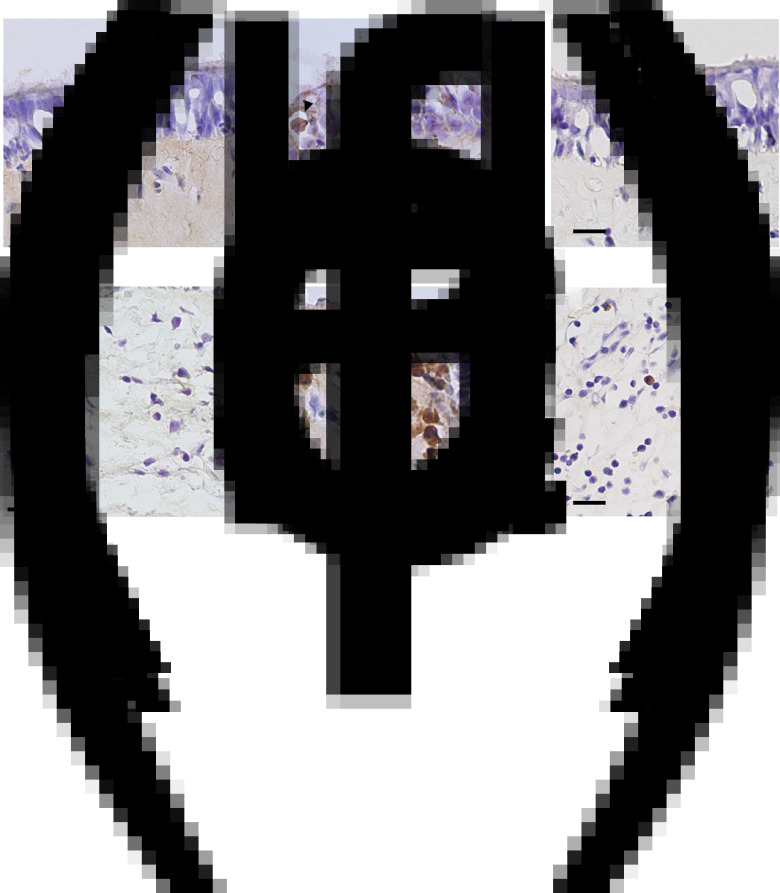
Representative immunohistological images showing angiotensin-converting enzyme-2 (ACE2) expression in ethmoid sinus mucosa (arrowheads) sampled from: a control subject (a & b), a non-eosinophilic chronic rhinosinusitis patient (c & d) and an eosinophilic chronic rhinosinusitis patient (e & f). Scale bars = 20 μm

Figure 3 shows representative histology from the three groups. The sinus mucosa of the control patients was covered with intact ciliated epithelia. Positive ACE2 immunoreactivity was localised mainly in the ciliated epithelial cells, especially on the apical surface including cilia (Figure 3a). Inflammatory cell infiltration was scarce in the control group, and less staining was observed in the submucosal layer (Figure 3b).

In the non-eosinophilic chronic rhinosinusitis group, enhanced ACE2 expression was observed, particularly in the damaged epithelial lining, where cell desquamation, focal cell loss and/or intra-epithelial mononuclear cell infiltration was present (Figure 3c). Intense ACE2 immunoreactivity was also detected in large mononuclear inflammatory cells, with cytoplasmic staining accumulated in the subepithelial layer (Figure 3d).

In contrast, the specimens from the eosinophilic chronic rhinosinusitis group generally showed less ACE immunoreactivity in the epithelial layer (Figure 3e). The eosinophilic chronic rhinosinusitis patients showed intense eosinophil infiltration in their ethmoid mucosa and nasal polyps on conventional histological examination, but less in the submucosal area compared to the non-eosinophilic chronic rhinosinusitis patients (Figure 3f).

## Discussion

Angiotensin-converting enzyme-2 is widely present in the heart, kidneys, lungs and testes, and it antagonises the activation of the classical renin–angiotensin system, suggesting an important role in homeostasis.^8^ Although it has been suggested that ACE2 may modulate inflammatory responses,^10,15^ its physiological role in the human respiratory tract is largely unknown. A protective effect of ACE2 in acute lung injury was reported in ACE2 knockout mouse models, with findings suggesting that treatment with recombinant ACE2 protein could reduce acute lung injury.^16^

Interleukin (IL)-13, but not IL-4 and IL-5, was negatively correlated with ACE2 and other T2-driven genes, which suggests an antagonistic relationship between ACE2 and type 2 inflammation with airway eosinophilia.^17^

Our study reports the pattern of ACE2 messenger RNA expression in the sinonasal mucosa of chronic rhinosinusitis patients and controls. The eosinophilic chronic rhinosinusitis patients had significant downregulation of ACE2 messenger RNA expression compared to the non-eosinophilic chronic rhinosinusitis and control subjects. This result is consistent with recent reports of a reduction in ACE2 and TMPRSS2 expression in patients with chronic rhinosinusitis with nasal polyps, compared to controls.^18^ In another report, ACE2 expression was reduced in the nasal and airway epithelial cells in patients with type 2 asthma and allergic rhinitis.^19^ The latter report speculated that the lower ACE2 expression in eosinophilic and allergic airway inflammation conditions indicated a potential regulatory effect.

We found that ACE2 messenger RNA levels were positively correlated with pro-inflammatory cytokines (tumour necrosis factor-α (TNF-α) and IL-1β) and negatively correlated with eosinophil-related chemokines (eotaxin-3) in chronic rhinosinusitis patients. The higher correlation with TNF-α may highlight the importance of host-microbe dynamics in sinus mucosa and/or imply a protective effect against viral infection. Tumour necrosis factor-α is a Th1-related cytokine and one of the most potent pro-inflammatory mediators.^20^ Tumour necrosis factor-α has pivotal roles in excessive mucus secretion with hyperplasia of goblet and glandular cells in type 1 inflammation, such as that found in non-eosinophilic chronic rhinosinusitis.^11,12,21^ In contrast, the presence of eosinophilic chronic rhinosinusitis is associated with typical type 2 inflammation and downregulation of ACE2 expression in paranasal sinus mucosa.

The distribution of ACE2 protein was localised mainly in the ciliated epithelial cells, especially on the apical surface. This is consistent with other reports which showed that ciliated cells and goblet or secretary cells in nasal mucosa expressed higher levels of ACE2 and TMPRSS2, and suggests that these cell types may be a portal of entry during SARS-CoV-2 infection.^6^

- Severe acute respiratory syndrome coronavirus-2 uses angiotensin-converting enzyme-2 (ACE2) as a primary receptor for transmission
- Angiotensin-converting enzyme-2 is highly expressed in human respiratory systems, including paranasal sinus mucosa
- Expression of ACE2 decreases in patients with eosinophilic chronic rhinosinusitis
- Levels of ACE2 expression are positively correlated with tumour necrosis factor-α and negatively correlated with eotaxin-3
- The dynamics of epithelial ACE2 expression depend on the different chronic rhinosinusitis types

The crown-like feature of coronaviruses is attributed to the presence of large type 1 transmembrane spike (S) glycoproteins. This S protein contains two distinct functional domains, S1 and S2.^22^ S1 contains the ACE2 receptor-binding domain, which facilitates viral attachment to the surface of target cells. The entry process is driven by the S2 subunit, which requires priming by a cellular serine protease (i.e. TMPRSS2). This allows the fusion of viral and cellular membranes.^23,24^ The modified S protein of SARS-CoV-2 has a 10- to 20-fold higher affinity for ACE2 than that of former SARS-CoV.^25,26^ This increased affinity may enable easier person-to-person spread of the virus.

Patients infected by SARS-CoV-2 have a range of respiratory symptoms and signs, including dry cough, fever, headache, dyspnoea, anosmia and hypogeusia, and pneumonia, with an estimated mortality rate in the range of 3–5 per cent.^2,3^ The major route of the viral transmission is respiratory droplets and direct contact, suggesting that the virus enters the host through the respiratory tract mucosa.^5^ In animal models, airway involvement was evident from the nasal turbinate to the trachea, with pulmonary alveoli associated with inflammatory changes and high viral load during the first week.^27^ We speculate that ACE2 expression in the sinonasal region may facilitate and regulate viral host-cell entry and replication in the epithelial cell layer.^28^

This study has some limitations. Its design was cross-sectional, and the sample collections were conducted prior to the Covid-19 pandemic. No data are available regarding the co-expression and distribution of TMPRSS2 (another key protein that is necessary for SARS-CoV-2 entry). Further research may elucidate functional roles of the paranasal sinus epithelial cells that are conditioned and primed to express these immune-associated genes and modify viral susceptibility. It remains to be clarified whether epithelial expression of ACE2 (the primary receptor for SARS-CoV-2 invasion) is influenced by host factors such as patient demographics or co-morbidities.^29^

## Conclusion

Epithelial expression of sinonasal ACE2 in chronic rhinosinusitis patients depends on the type of inflammatory responses present. Patients with eosinophilic chronic rhinosinusitis had a significant downregulation of ACE2 messenger RNA expression compared to the non-eosinophilic chronic rhinosinusitis patients and control subjects. The apical expression of ACE2 on epithelial cells posits that this virus receptor is accessible as a portal for infection. These findings may contribute to the understanding of SARS-CoV-2 transmission, and serve as a useful area for future research into prevention and control of this virulent pathogen.
